# Analysis of bortezomib inhibitor docked within the catalytic subunits of the *Plasmodium falciparum* 20S proteasome

**DOI:** 10.1186/2193-1801-2-566

**Published:** 2013-10-26

**Authors:** Settu Sridhar, Gayathri Bhat, Kunchur Guruprasad

**Affiliations:** Bioinformatics, Centre for Cellular and Molecular Biology, Hyderabad, 500 007 India

**Keywords:** *Plasmodium falciparum*, 20S proteasome, Catalytic sites, Bortezomib inhibitor, Binding pockets, Plasmodia-specific insert

## Abstract

The three-dimensional fold of *Plasmodium falciparum* (Pf) 20S proteasome is similar to yeast *Saccharomyces cerevisiae* 20S proteasome. The twenty eight subunits complex corresponding to two copies of seven distinct α and seven distinct β subunits shares >35% sequence identity with equivalent subunits of the yeast 20S proteasome. Bortezomib (Velcade®) – a known inhibitor of the three catalytic subunits; β1, β2, β5 of the yeast 20S proteasome can bind in the equivalent subunits of the Pf 20S proteasome and is in agreement with experimental results. The model defines the binding mode of the bortezomib inhibitor within the catalytic subunits of the Pf 20S proteasome and provides the structural basis for the design of Pf 20S proteasome-specific inhibitors. The substitutions associated within the catalytic subunits of Pf 20S proteasome relative to yeast 20S proteasome; Thr21-Ser, Thr22-Ser, Thr31-Ser, Thr35-Asn, Ala49-Ser (in β1 subunit), Ser20-Ala, Gln22-Glu (β2) and Thr21-Ser, Ala22-Met, Gln53-Leu (β5) may influence the relative caspase-like, tryptic-like and chymotryptic-like activities of the Pf 20S proteasome. The plasmodia-specific 'large’ insert comprising fifty four amino acid residues (in β1 subunit) of the Pf 20S proteasome is distant from the catalytic sites.

## Introduction

An essential element of the protein quality control machinery in cells is the ubiquitin-proteasomal system (Hershko & Ciechanover 1998; Pickart 2001; Myung et al. 2001). Proteasomes (Wolf & Hilt 2004) are protein degradative machines found in the nucleus and cytoplasm of all eukaryotic organisms and archaebacteria and is a highly organized protease complex comprising a catalytic 20S core particle (CP) and two 19S regulatory particles (RP), which together form the 26S structure. The core particle degrading machinery in yeast *Saccharomyces cerevisiae* 20S proteasome is coded by fourteen genes and two copies of these constitute the twenty eight subunits in the complex. The 26S proteasome multi-subunits complex is the final destination for selective degradation of majority of cellular proteins and is responsible for the degradation of most ubiquitylated proteins through a multistep process involving recognition of the polyubiquitin chain, unfolding of the substrate, and translocation of the substrate into the active site in the cavity of the CP. The structure, function, assembly and catalytic mechanism of the proteasome are reviewed in (Jung & Grune 2012; Saeki & Tanaka 2012; Coux et al. 1996; Marques et al. 2009). The crystal structures of yeast 20S proteaseome (Groll et al. 1997) and bovine 20S proteasome (Unno et al. 2002) are available in the Protein Data Bank (PDB) (Rose et al. 2013). The crystal structure of the core particle in eukaryotic yeast *Saccharomyces cerevisiae*, has a barrel-shaped cylindrical structure that is comprised of four heptameric rings (α_1-7_β_1-7_β_1-7_α_1-7_), in which each subunit is different from the other and individual subunits are uniquely located in the three-dimensional structure of the complex (Groll et al. 1997). The α-ring on the outer sides functions as a gate for the substrate to enter while the β-ring has the proteolytic activity. In yeast 20S proteasome, the β1, β2 and β5 subunits are known to possess catalytic activity, where β1 subunit mediates the peptidyl-glutamyl peptide hydrolyzing post-acidic (caspase-like) activity; β2 subunit the post-basic (trypsin-like) activity and the β5 subunit post-hydrophobic (chymotrypsin-like) activity (Arendt & Hochstrasser 1997; Heinemeyer et al. 1997). The proteasomes share the fold and a novel catalytic mechanism with an N-terminal nucleophilic threonine and therefore placed in the family of Ntn (N terminal nucleophile) hydrolases (Bochtler et al. 1999). The crystal structure of bovine 20S proteasome revealed an additional novel catalytic subunit – the β7 subunit (Unno et al. 2002). In addition to the 20S core proteasome, the yeast 26S proteasome comprises two regulatory subcomplexes containing nineteen subunits (referred as 19S regulatory complex); six Rpt (Regulatory particle ATPases) and thirteen RPN (Regulatory particle Non-Atpases) attached at terminal ends of the central portion and in opposite orientations (DeMartino & Slaughter 1999; Voges et al. 1999). The complete subunit architecture of the yeast regulatory particle provides a model for the recognition, deubiquitination and engagement of a polyubiquitinated substrate by the 26S proteasome (Lander et al. 2012). The molecular architecture of the 26S holocomplex has been determined by an integrative approach based on data from cryoelectron microscopy, X-ray crystallography, residue-specific chemical cross-linking, and several proteomic techniques (Lasker et al. 2012; Förster et al. 2010; Nickell et al. 2009). Proteasomes are central to many cellular processes as they are responsible for the cytoplasmic turnover of a number of proteins and therefore manipulating the proteasomal activity is considered a key goal in controlling the stability of regulatory proteins (Groll & Huber 2004; Kisselev & Goldberg 2001). The observation that proteasome inhibitors cause apoptosis in certain tumor-derived cell lines has led to their application as potential cancer therapeutics (Adams et al. 1998). Bortezomib (Velcade®) has been approved for treatment of multiple myeloma patients (Ludwig et al. 2005; Richardson et al. 2005; Teicher et al. 1999). The proteasome is a potential target for treating many infections and diseases (Dahlmann 2007).

In plasmodia two T1 threonine peptidase systems are known to be present; the 20S proteasome is enzymatically active and expressed throughout the live cycle, whereas the PfhsIV is expressed in late stages of development only (Mordmüller et al. 2006). The emergence and spread of *Plasmodium falciparum* resistance to almost all available antimalarial drugs has necessitated the search for new chemotherapeutic compounds. It has been shown that the 20S proteasome is expressed and catalytically active in plasmodia and treatment with proteasome inhibitors arrests parasite growth and therefore inhibition of the proteasome is considered to be a highly promising strategy to develop new antimalarials (Kreidenweiss et al. 2008).

A three-dimensional model of the twenty eight subunits complex corresponding to the core particle (CP) of *Plasmodium falciparum* (Pf) is currently not available, although individual models for twelve of the fourteen genes of the CP are available in the ModBase database (Pieper et al. 2011). Bortezomib, a peptide boronate, is the only proteasome inhibitor in clinical use so far. In contrast to multiple myeloma treatment (Rajkumar et al. 2005), its activity in *P. falciparum* laboratory strains is low (Kreidenweiss et al. 2008). The crystal structure of yeast 20S proteasome bound to bortezomib (Groll et al. 2006) is available in the PDB. Therefore, in order to evaluate the mode and affinity of binding of the bortezomib inhibitor within the equivalent catalytic subunits of the Pf 20S proteasome, we have constructed a three-dimensional model based on the crystal structures of the homologous yeast and bovine 20S proteasomes and docked bortezomib within the catalytic subunits of the Pf 20S proteasome model. Further, we have identified substitutions within the catalytic subunits of the Pf 20S proteasome relative to the yeast 20S proteasome. Our models of the Pf 20S proteasome complexed with the bortezomib inhibitor provide a structural basis for further design of Pf 20S proteasome-specific inhibitors that has implications for the treatment of malaria.

## Materials and methods

### Selection and identification of Pf 20S proteasome subunit sequences

The Pf proteasome subunit sequence codes were selected from the PlasmoDB database (Aurrecoechea et al. 2009). Their equivalent codes from the UniProt database (The UniProt Consortium 2010) were also identified. The homologs of these subunits corresponding to proteins of known three-dimensional structure were identified from the PDB using the PSI-BLAST program (Altschul et al. 1997).

### Construction & validation of the Pf 20S proteasome and docking of bortezomib within the catalytic subunits

The crystal structures of the yeast 20S proteasome (PDB code:1RYP) (Groll et al. 1997) and bovine 20S proteasome (PDB code:1IRU) (Unno et al. 2002) were used as templates in the comparative protein modeling software program MODELER (Eswar et al. 2008) for constructing a three-dimensional model of the Pf 20S proteasome. The individual Pf 20S proteasome subunit sequences were aligned along with their equivalent sequences in yeast and bovine 20S proteasomes. MODELER constructs a 3-D model for the query sequence using sequence-to-template alignment and the satisfaction of spatial restraints derived from the template structure(s) (Sali & Blundell 1993). The overall quality of the protein model was evaluated using the PROCHECK program (Laswoski et al. 1993). All pictures were generated using PyMolhttp://sourceforge.net/projects/pymol/. The docking of bortezomib inhibitor was carried out using AutoDock (Morris et al. 2009). In order to validate our docking studies, the coordinates of bortezomib was removed from the crystal structure of the yeast 20S proteasome (PDB code: 2F16). A model of the yeast 20S proteasome with bortezomib docked within the catalytic subunits using AutoDock was generated. The docked complex was structurally superimposed on to the crystal structure complex of yeast 20S proteasome with bound bortezomib in the three catalytic subunits; β1, β2 and β5 (PDB code: 2F16). The binding mode of bortezomib and inter-molecular interaction energies within the three catalytic subunits of the yeast 20S proteasome were evaluated. Upon successfully reproducing the bortezomib binding in the yeast 20S proteasome, bortezomib was then docked in the equivalent catalytic subunits of the Pf 20S proteasome model. The binding mode and intermolecular interaction energies were once again evaluated and compared with results obtained for bortezomib binding in the yeast 20S proteasome.

### Comparison of the catalytic sites, substrate binding pockets and bortezomib binding in 3-D models of Pf 20S proteasome and crystal structure complex of yeast 20S proteasome

The residues important for the catalytic activity, substrate binding pockets and maintenance of stability of the conformation of Thr1 via hydrogen bond essential for the Ntn hydrolases was obtained from the crystal structure of the yeast 20S proteasome (PDB code: 1RYP). Further, the interactions made by the bortezomib inhibitor in the catalytic subunits of the yeast 20S proteasome were obtained from (Groll et al. 2006) and LigPlot diagrams (Wallace et al. 1995) for the crystal structure of the yeast 20S proteasome with bortezomib inhibitor (PDB code:2F16). The amino acid sequences corresponding to the individual catalytic subunits from yeast, bovine and Pf 20S proteasomes were aligned using the CLUSTALW program (Thompson et al. 1994). The molecular graphics software spdview (Guex & Peitsch 1997) was used to generate the hydrogen-bond interactions between the bortezomib inhibitor and residues in the binding pockets of the yeast and Pf 20S proteasomes.

## Results and discussion

The accession codes corresponding to the individual subunit sequences of Pf 20S proteasome are shown in Table 1. The homologous chains in yeast and bovine 20S proteasomes and the ModBase IDs corresponding to 3-D models available for subunits are listed.

**Table 1:** ***Plasmodium falciparum*** **20S proteasome subunit sequences, UniProt accession codes and equivalent PlasmoDB IDs**

S.No.	UniProt accession codes for Pf20S proteasome sequences	PDB chains corresponding to yeast homologous subunits for Pf 20S proteasome sequences	PDB chains corresponding to bovine homologous subunits for Pf 20S proteasome sequences	PlasmoDB_ID for Pf 20S proteasome subunits	ModBase_ID for Pf 20S proteasome subunit 3-D models
1	Q8I261	1RYP/J/X	1IRU/J/X	PF3D7_0108000	PFA0400c 3202
2	Q8IDG3	1RYP/C/Q	1IRU/C/Q	PF3D7_1353800	No model
3	Q8IDG2	1RYP/D/R	1 IRU /D/R	PF3D7_1353900	MAL13P1.270 185
4	Q8IJT1	1RYP/L/Z	1 IRU /L/Z	PF3D7_1011400	PF10_0111 1187
5	Q7K6A9	1RYP/N/2	1 IRU /N/2	PF3D7_0803800	MAL8P1.142 660
6	C6KST3	1RYP/B/P	1 IRU /B/P	PF3D7_0608500	TR Q7RK69
7	Q8I6T3	1RYP/I/W	1 IRU /I/W	PF3D7_1328100	PF13_0156 2140
8	Q8IBI3	1RYP/E/S	1 IRU /E/S	PF3D7_0727400	PF07_0112 912
9	Q8IK90	1RYP/F/T	1 IRU /F/T	PF3D7_1474800	PF14_0716 3046
10	Q8IKC9	1RYP/K/Y	1 IRU /K/Y	PF3D7_1470900	No_ID
11	Q8IAR3	1RYP/A/O	1 IRU /A/O	PF3D7_0807500	No_ID
12	C0H4E8	1RYP/M/1	1 IRU /M/1	PF3D7_0518300	No_ID
13	Q8I0U7	1RYP/H/V	1 IRU /H/V	PF3D7_0931800	No model
14	O77396	1RYP/G/U	1 IRU /G/U	PF3D7_0317000	PFC0745c 3667

PDB chains corresponding to homologs of yeast and bovine 20S proteasome subunits and the ModBase IDs for individual Pf 20S proteasomal subunit models.

The Pf 20S proteasome subunit sequences share > 35% sequence identity with equivalent subunits in yeast and bovine 20S proteasomes. Certain regions excluded in the model and listed in Table 2, mainly correspond to the N-terminal regions of the catalytic β-subunits. These regions are processed at the Gly-Thr1 site during the formation of the active core proteasome. Further, the β1 and β7 subunits comprise certain 'large’ insertions in the middle of the subunit sequences and these were also excluded in the model due to the lack of suitable templates for modeling. The 'large’ insertions of fifty four and thirty five amino acid residues are associated with the subunits (UniProt codes: Q8I0U7 and Q7K6A9, respectively).

**Table 2:** **Excluded regions in the Pf 20S model and their corresponding subunits**

S. No.	Pf 20S proteasome subunit sequences UniProt accession code	Excluded regions in the model (location)	Number of amino acid residues
1	Q8IJT1	MVIAS…..DFHKG (N-ter)	60
2	Q7K6A9	NSQKYD…..EYKEI (middle)	35
3	Q8I6T3	MKLEY…..FRKTG (N-ter)	41
4	Q8IBI3	MFSTRSEY (N-ter)	8
		IDMTA (C-ter)	5
5	Q8IAR3	MVRPSQ (N-ter)	6
6	C0H4E8	MDLIL.....GRGFK (N-ter)	28
7	Q8I0U7	MDVVN.....TPISD (N-ter)	29
		KGRFH…..KFNDY (middle)	53

### Three-dimensional model of the Pf 20S proteasome and the Plasmodia-specific 'large’ insert sequences in some of the beta subunits

**Figure 1:**
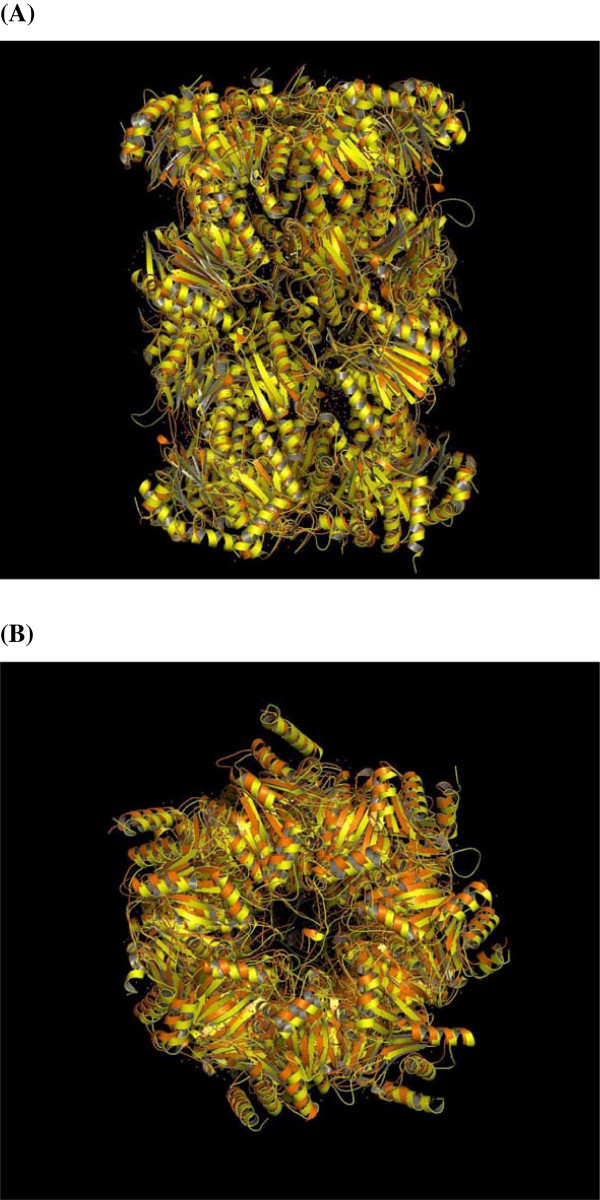
**Schematic representation showing structural overlay of the crystal structure of yeast 20S proteasome [PDB code:1RYP] (orange) and the Pf 20S proteasome model (yellow); (A) side-view and (B) top-view.**

The quality of the Pf 20S proteasome model evaluated using the PROCHECK program for the individual subunits identified >90% residues in the 'allowed’ regions of the Ramachandran Map suggesting the models are of good quality. A cartoon representation of the Pf 20S model compared with the crystal structure of the yeast 20S proteasome is shown in Figure 1(A). The overall fold of the CP is similar to that of the yeast 20S proteasome. The cavity on the side of the α-ring where the substrate enters the catalytic site is shown in Figure 1(B).

**Figure 2:**
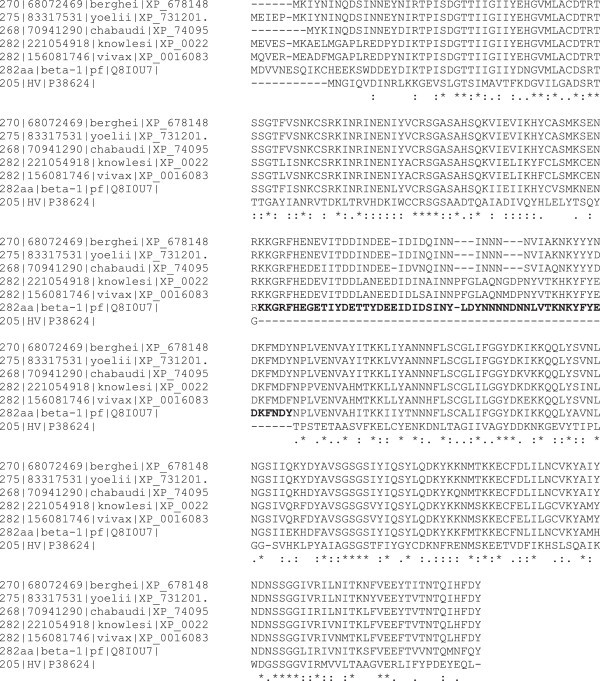
**Multiple sequence alignment corresponding to the β1 subunit sequences in** ***Plasmodium falciparum*** **and 5 other** ***Plasmodium*** **species and the yeast 20S proteasome.** The 'large’ insert region is common to the 20S proteasomes of the *Plasmodium* species analyzed. '*’ indicates identical residues, ':’ indicates conservative substitutions, '.’ indicates >50% of residues are conserved in the alignment.

The 'large’ inserts are known to correspond to coding regions of low complexity (LCRs) which are a feature of many proteins in plasmodia. Although, the structure of these inserts are not known, its function has been suggested in the obstruction of immune-dominant epitopes and the presentation of 'non-sense’ epitopes that lead to an inefficient antibody response (Mordmüller et al. 2006). Our model of the Pf 20S proteasome suggests that these 'large’ inserts are distant from the catalytic sites and are not likely to interfere with catalytic activity. Our observations are consistent with earlier predictions that suggest that the structural integrity and protease function seem not to be affected due to the 'large’ inserts (Mordmüller et al. 2006). Experimental studies aimed at truncating the 'large’ inserts and re-design of the β1 and β7 subunits of the Pf 20S proteasome guided by the equivalent yeast 20S proteasome subunits may provide clues to decipher their role in the Pf 20S proteasome. Examination of the sequences corresponding to the β1 subunit from other plasmodia species reveals that the 'large’ insert is a feature common to all plasmodia species analyzed in this work. The alignment of the corresponding sequences is shown in Figure 2.

### Comparison of the catalytic residues, substrate binding pockets and residues involved in maintaining stability of the conformation of Thr1 in yeast and Pf 20S proteasomes

**Figure 3:**
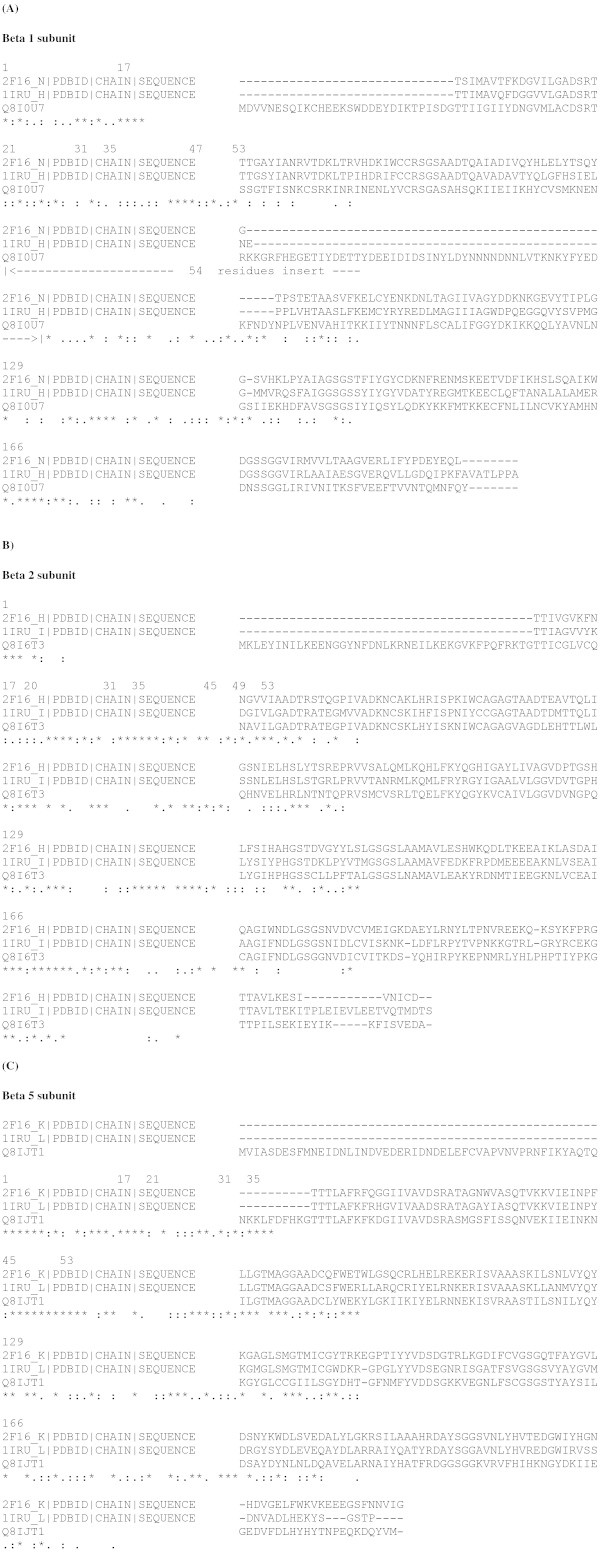
**Multiple sequence alignment corresponding to (A) β1, (B) β2 and (C) β5 subunit sequences of the yeast 20S proteasome [PDB code:2F16], bovine 20S proteasome [PDB code:1IRU] and Pf 20S proteasome [UniProt codes].** Residue labeling is according to PDB code:2F16.

The crystal structure of yeast 20S proteasome (Groll et al. 1997) revealed that the amino acid residues; Thr1, Asp17 and Lys33 are important for catalytic activity, Ser129, Asp166, Ser169 are important for maintaining stability of the Thr1 conformation and amino acid residues at positions; 1, 20, 31, 35, 45, 49 and 53 comprise the substrate binding pockets for proteolytic cleavage in the β1, β2 and β5 catalytic subunits. The alignment of the amino acid sequences corresponding to the equivalent β1, β2 and β5 catalytic subunits in the Pf 20S proteasome relative to yeast and bovine 20S proteasomes are shown in Figures 3(A-C) and labeled according to the numbering in the yeast 20S proteasome crystal structure (PDB code: 2F16).

The amino acid residues in the different catalytic subunits of the yeast 20S proteasome and the equivalent residues (substitutions) in the Pf 20S proteasome are listed in Table 3.

**Table 3:** **Amino acid residues in β1, β2, β5 catalytic subunits in yeast 20S proteasome (PDB code: 2F16:N,H,K-chains, respectively) and equivalent residues (substitutions) in Pf 20S proteasome associated with the catalytic sites, substrate binding pockets, maintenance of stability of Thr1 and interactions with the bortezomib inhibitor**

Residue Number	Catalytic subunits		
	β1	β2	β5
1	T	T	T
17	D	D	D
20	T	S(A)	A
21	T(S)	T	T(S)
22	T(S)	Q(E)	A(M)
31	T(S)	C	V
33	K	K	K
35	T(N)	H	I
45	R	G	M
47	G	G	G
49	A(S)	A	A
53	Q	E	Q(L)
129	S	S	S
166	D	D	D
169	S	S	S

The amino acid residues important for the catalytic activity in yeast 20S proteasome and associated with the β1 (PDB code: 2F16_N), β2 (2F16_H) and β5 (2F16_K) subunits are also present in the equivalent Pf 20S proteasome subunits, suggesting their highly conserved nature and that these equivalent subunits in the Pf 20S proteasome would also be associated with catalytic activity. The catalytic subunits in yeast 20S proteasome are associated with P1 cleavage sites of chromogenic reporter groups, peptidylglutamyl-peptide hydrolytic (PGPH) or post-acidic (β1), trypsin-like or post-basic (β2), chymotrypsin-like or post-hydrophobic (β5) activities (Arendt & Hochstrasser 1997). The substitutions observed in the corresponding subunits of the Pf 20S proteasome at positions; 22, 31 and 35 and certain additional substitutions in some of the subunits at positions; 20, 21, 49, 53 are likely to contribute to the substrate specificity of the Pf 20S proteasome relative to yeast 20S proteasome. The Pf 20S proteasome also contains the novel catalytic β7 subunit identified in bovine 20S proteasome (Unno et al. 2002) as it is characterized by the conserved N-terminal Gly-Thr1 residues observed in the other catalytic subunits.

### Comparative analysis of interactions of the bortezomib inhibitor in the three catalytic subunits of Pf 20S proteasome model and the crystal structure of yeast 20S proteasome

**Figure 4:**
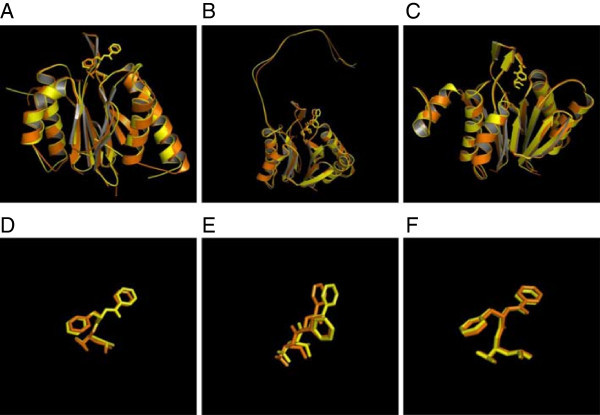
**Schematic representations of the structural overlay corresponding to the crystal structure of the yeast 20S proteasome bortezomib inhibitor complex [PDB code:2F16] (orange) and the model of the Pf 20S proteasome with docked bortezomib inhibitor (yellow) in the catalytic sites of (A) β1, (B) β2 and (C) β5 subunits.** Magnified images of the bortezomib inhibitors (in stick representation) are shown for the above subunits in **(D)**, **(E)** and **(F)**, respectively.

The crystal structure complex of yeast 20S proteasome with the known inhibitor – bortezomib (N-[(1R)-1-(dihydroxyboryl)-3-methylbutyl]-N-(pyrazin-2-ylcarbonyl)-L-phenylalaninamide) (PDB code:2F16) in the three catalytic sites; β1, β2 and β5 provided the structural basis at atomic resolution for the different in vivo binding affinities of bortezomib for the individual subunits that are roughly attributed to the interactions of the leucine, pyrazine and boronate moieties (Groll et al. 2006). Our docking results were able to successfully reproduce the binding mode of bortezomib as observed in the crystal structure of the yeast 20S proteasome-bortezomib complex (PDB code: 2F16). The structural overlay showing the cartoon representation of the crystal structure of yeast 20S proteasome with bortezomib inhibitor (orange) (PDB code: 2F16) and bortezomib docked in the three-dimensional model of the Pf 20S proteasome (yellow) corresponding to the β1, β2 and β5 subunits are shown in Figures 4[A,B,C], respectively. These figures demonstrate the overall structural similarity of the catalytic subunits in yeast and Pf 20S proteasomes and the similar binding modes of bortezomib in the equivalent subunits. The magnified images of the docked bortezomib inhibitor (Figures 4D, E and F) shows its binding mode in the β1, β2 and β5 subunits, respectively. The β2 subunit of the Pf 20S proteasome is associated with a relative displacement of the pyrazine moiety of bortezomib (Figure 4E) compared to that in yeast 20S proteasome.

The intermolecular interaction energies obtained from AutoDock for binding of bortezomib within the equivalent catalytic subunits of yeast and Pf 20S proteasomes are shown in Table 4.

**Table 4:** **Intermolecular interaction energies of Bortezomib (Velcade®) inhibitor in the catalytic subunits of the crystal structure of yeast 20S proteasome and in the equivalent subunits of the Pf 20S proteasome model**

Catalytic subunits	Intermolecular Energy (kcal/mol)
2F16_Beta1	-6.45
2F16_Beta2	-6.60
2F16_Beta5	-6.84
Pf_Beta1	-5.84
Pf_Beta2	-1.54
Pf_Beta5	-4.29

**Figure 5:**
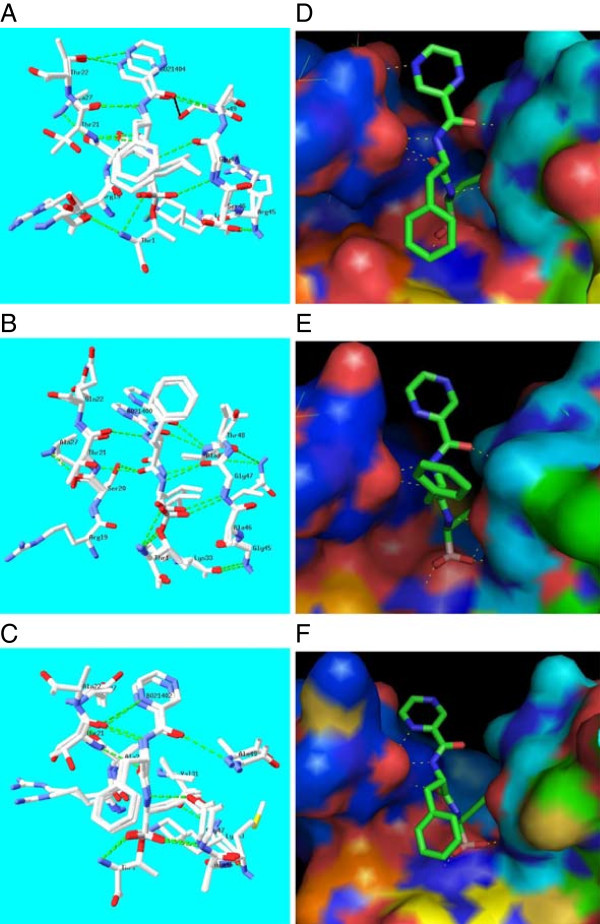
**Structural superposition of the yeast 20S proteasome - bortezomib inhibitor crystal structure complex [PDB code:2F16] and Pf 20S proteasome – bortezomib model showing inter-molecular hydrogen bond interactions and residues close to the inhibitor defined by a 4.0 Å cut-off value in (A) β1, (B) β2 and (C) β5 subunits.** The electrostatic charge surface (red;electronegative, blue;electropositive) for the Pf 20S proteasome model with bound bortezomib for the above subunits are shown in figures **(D)**, **(E)** and **(F)**, respectively.

Despite the observed substitutions in the equivalent catalytic subunits of the Pf 20S proteasome relative to yeast 20S proteasome, most hydrogen bond interactions between bortezomib and yeast 20S proteasome are also present in the models of the Pf 20S proteasome-bortezomib complex as shown in Figures 5(A-C).

In the β1 subunit of the yeast 20S proteasome (PDB code:2F16, N-chain), the bortezomib inhibitor (BO2 1404) makes hydrogen bond interactions with residues in the binding pockets involving the main-chain atoms of Thr21, Gly47, Ala49 and side-chain atom of Thr22 and both main-chain and side-chain atoms of Thr1 (Groll et al. 2006). By comparison, all the above interactions are also observed in the corresponding β1 subunit of the Pf 20S proteasome (Figure 5A). The equivalent hydrogen-bond interactions with bortezomib at positions 21 and 22 are also conserved despite the substitutions; Thr21-Ser, Thr22-Ser, as serine only lacks the side-chain C-gamma atom compared with threonine. The Ala49-Ser mutation contributes an extra hydrogen-bond interaction via the side-chain OG atom of serine in the Pf 20S proteasome β1 subunit relative to yeast 20S proteasome favoring better binding interactions to bortezomib.

In the β2 subunit of the yeast 20S proteasome (PDB code:2F16, H-chain), bortezomib (BO2 1400) makes hydrogen bond interactions with residues in the binding site pockets involving the main-chain atoms of Thr21, Gly47, Ala49 and side-chain atom of Ser20 and both the main-chain and side-chain atoms of Thr1 (Groll et al. 2006). All these interactions are also observed in the corresponding β2 subunit of the Pf 20S proteasome except the side-chain hydrogen-bond interaction with bortezomib due to the Ser20-Ala substitution (Figure 5B). Further, the side-chain hydrogen bond interactions made by Thr22 in the β1 subunit of yeast 20S proteasome is not observed at the equivalent position in the β2 subunit of both yeast and Pf 20S proteasomes due to substitutions by Gln22 and Glu22, respectively. For these reasons, the pyrazine moiety of bortezomib is not likely to make any interaction with protein residues in the β2 subunits of both yeast and Pf 20S proteasomes. Further, the Ser20Ala substitution may contribute to the relative displacement (weaker binding) of bortezomib in the β2 subunit of Pf 20S proteasome compared to that in the yeast 20S proteasome (see Figures 4E and 5E). The phenylalanine ring is 'flipped’ relative to the other two catalytic subunits and it has been suggested that the P2 site of bortezomib possibly contributes to overall pharmacodynamic properties, but not kinetics of inhibition (Groll et al. 2006). In case of the β2 subunit of Pf 20S proteasome too, the phenylalanine moiety is exposed to the surface and is 'flipped’ relative to its conformation in the β1 and β5 catalytic subunits as observed in the β2 subunit of the yeast 20S proteasome. The binding affinity of bortezomib in the β2 subunit of Pf 20S proteasome may therefore be lower compared to its binding in the β2 subunit of the yeast 20S proteasome as reflected in the relatively higher intermolecular energies for bortezomib binding to the β2 subunit of Pf 20S proteasome shown in Table 4. Moreover, it is also known that bortezomib has least preference for the β2 subunit in the yeast 20S proteasome (Berkers et al. 2005). The Glu22 in β2 subunit of the Pf 20S proteasome has two oxygen atoms (OE1, OE2) in its side-chain when compared with Gln22 in yeast 20S proteasome that may be exploited in designing suitable bortezomib analogs that make favourable interaction with the inhibitor in the β2 subunit of Pf 20S proteasome.

Finally, in the β5 subunit of the yeast 20S proteasome (PDB code:2F16, K-chain), bortezomib (BO2 1402) makes hydrogen bond interactions with residues in the binding pockets involving the main-chain Thr21, Gly47, Ala49 and main-chain and side-chain of Thr1 (Groll et al. 2006). These interactions are also observed in the corresponding β5 subunit of the Pf 20S proteasome (Figure 5C). The Thr21-Ser substitution in the β5 subunit of the Pf 20S proteasome does not affect the hydrogen-bond interactions with bortezomib. All other residues comprising the inhibitor binding pocket remaining the same, the substitutions; Ala22-Met, Gln53-Leu in the β5 subunit of the Pf 20S proteasome may affect the relative chymotryptic-like activity compared with the yeast 20S proteasome.

The preference for the affinity of bortezomib to the β5 subunit relative to the other two catalytic subunits in yeast 20S proteasome, i.e., β5 > β1 > > β2 (Berkers et al. 2005) is reflected in the low intermolecular interaction energy for the β5 subunit (-6.84 kcal/mol) as shown in Table 4. In Pf 20S proteasome, however, bortezomib appears to have a preference for the β1 subunit as inferred from the relative intermolecular interaction energies. The observed substitutions in the β1 and β5 subunits of the Pf 20S proteasome compared to the yeast 20S proteasome mentioned earlier may contribute to the relative preference for bortezomib inhibition. Accordingly, bortezomib inhibition may influence the caspase-like and chymotryptic-like activities in Pf 20S proteasome compared to that in yeast 20S proteasome. The binding affinity of bortezomib to the β2 subunit of the Pf 20S proteasome is least as inferred from the relative high intermolecular interaction energy value (-1.54 kcal/mol) possibly due to the loss of side-chain hydrogen-bond interactions in the β2 subunit of the Pf 20S proteasome due to Ser20Ala substitution.

In an earlier comprehensive study on the proteasome inhibitors against *Plasmodium falciparum* laboratory strains, bortezomib inhibitory activity was observed to be low (Kreidenweiss et al. 2008) in contrast to multiple myeloma treatment (Rajkumar et al. 2005). Our analysis is in agreement with the above experimental observations. Bortezomib is capable of binding to the equivalent catalytic subunits of the Pf 20S proteasome although relatively less efficiently as reflected in the relatively higher intermolecular energy values for the Pf 20S proteasome shown in Table 4. The lower efficiency of bortezomib binding in the catalytic subunits of Pf 20S proteasome relative to yeast 20S proteasome may be due to the observed substitutions within the bortezomib binding pockets. Also, the substitutions described for the Pf 20S proteasome contribute to the different charge patterns and overall architecture (see Figures 5D, E and F) as observed in the yeast 20S proteasome (Groll et al. 2006).

The binding mode of bortezomib inhibitor is similar to that observed in the catalytic subunits of the yeast 20S proteasome, except in the β2 subunit of the Pf 20S proteasome where there is a relative displacement due to the observed substitution. Therefore, the substitutions identified in the equivalent catalytic subunits of the Pf 20S proteasome compared to the yeast 20S proteasome may only affect the relative caspase-like, tryptic-like and chymotryptic-like activities although bortezomib binding may not be largely affected. The phenylalanine moiety of bortezomib is exposed and does not make interactions with the protein subunits also in the Pf 20S proteasome as is observed in the yeast 20S proteasome. Our models of the Pf 20S proteasome with bortezomib provide the structural basis for rational design of Pf 20S proteasome specific inhibitors.

We suggest other possible strategies for Pf 20S proteasome inhibition, such as, to design suitable compounds that block interactions at the interface of α/β, α/α or β/β subunits, as it is understood that the α and β subunits are formed first with subsequent formation of the seven membered ring (Wolf & Hilt 2004). Further, the formation of the active 20S proteasome is preceded by the cleavage of its pro-sequence. Therefore, the structure of the inactive catalytic subunit with the pro-sequence bound may provide clues to designing suitable inhibitors. As high resolution structural data for the regulatory subunits become available, it would provide further opportunities for the design of compounds that inhibit substrate recognition or molecular events that guide the entry of the substrate towards the catalytic core. The availability of a number of other proteasome inhibitors (Kreidenweiss et al. 2008; Buac et al. 2013) provides an opportunity to carry out docking studies with these compounds in the three-dimensional models of the Pf 20S proteasome that are relevant for malaria therapeutics.

Finally, another observation that we have made, although not related to the Pf 20S proteasome catalytic sites is the comparison of sequences of the 20S proteasomes of bovine, yeast and Pf with regard to the Nuclear Localization Signal (NLS). The bovine 20S proteasome contains a sequence motif [X-X-K-K(R)-X-K(R)] associated with the α1 - α4 subunits needed for its import into nucleus by the importin-α receptor (Unno et al. 2002). This sequence motif is not present in the equivalent α subunits of the Pf and yeast 20S proteasomes as shown in Table 5.

**Table 5:** **Nuclear localization signal [X-X-K-K(R)-X-K(R)] in bovine 20S proteasome and equivalent amino acid sequence in Pf and yeast 20S proteasomes**

Alpha subunits	Bovine	Pf	Yeast
1	LE**KK**V**K**(179–184)	LERLLE	LENHFK
2	TE**KK**Q**K**(47–52)	TEKKSP	TEKKSS
3	RE**KK**E**K**(249–254)	------	EEADED
4	EK**KK**Q**K**(240–245)	NEQNE-	QEQDKK

## Conclusions

The three-dimensional model of the Pf 20S proteasome complexed with the bortezomib inhibitor supports previous experimental studies that demonstrate bortezomib inhibition of the Pf 20S proteasome and further elucidates its binding mode within the catalytic subunits. The affinity for bortezomib binding to the Pf laboratory strains is known to be low in contrast to binding eukaryotic 20S proteasome as evinced from multiple myeloma treatment and the relative intermolecular interaction energies obtained from our docking studies is consistent with the above results. Further, our docking analysis suggests a relative preference for bortezomib binding to the β1 subunit in the Pf 20S proteasome. The amino acid residue substitutions identified within the catalytic subunits are useful candidates for evaluating the structure-activity relationship of the Pf 20S proteasome. Our models provide the structural basis for rational design of Pf 20S proteasome-specific catalytic site inhibitory compounds among other strategies that exploit structural information for the purpose of drug design.
